# Complete Genome Sequence of the Neonatal Meningitis Escherichia coli Serotype O18:K1 Strain NMEC15

**DOI:** 10.1128/MRA.00832-21

**Published:** 2021-09-23

**Authors:** Aline L. de Oliveira, Timothy J. Johnson, Nicolle L. Barbieri, Lisa K. Nolan, Catherine M. Logue

**Affiliations:** a Department of Microbiology, University of Georgia, Athens, Georgia, USA; b Department of Veterinary and Biomedical Sciences, College of Veterinary Medicine, University of Minnesota, Saint Paul, Minnesota, USA; c Department of Population Health, College of Veterinary Medicine, University of Georgia, Athens, Georgia, USA; d Department of Infectious Diseases, College of Veterinary Medicine, University of Georgia, Athens, Georgia, USA; University of Maryland School of Medicine

## Abstract

Neonatal meningitis Escherichia coli (NMEC) is the second leading cause of sepsis and meningitis in neonates worldwide. Here, we report the genome sequence of NMEC15, belonging to serotype O18:K1, isolated from the cerebrospinal fluid (CSF) of an infant with neonatal bacterial meningitis (NBM) in the Netherlands.

## ANNOUNCEMENT

Neonatal meningitis Escherichia coli (NMEC) is the second leading cause of sepsis and meningitis in neonates worldwide (1). However, NMEC has emerged as the most common cause of meningitis and sepsis among very-low-birth-weight infants (<1,500 g birth weight) since the 1990s (2–5).

NMEC15 (serotype O18:K1) was isolated from the cerebrospinal fluid (CSF) of a newborn infant (<28 days) with meningitis in the Netherlands as described elsewhere (6, 7). The O serogroup was identified at the E. coli Reference Center at Pennsylvania State University. NMEC15 belongs to serotype O18:K1, similar to the prototypic NMEC strain RS218 (8). Here, we present the genome sequence of NMEC15.

NMEC15 was grown on LB agar and subsequently in Luria-Bertani broth at 37°C. Genomic DNA (gDNA) was extracted using the ChargeSwitch gDNA mini bacteria kit (Life Technologies, Carlsbad, CA) for Illumina sequencing. The DNA yields were quantified using a Qubit fluorimeter double-stranded DNA (dsDNA) high-sensitivity (HS) kit (Life Technologies). The QIAseq FX kit (Qiagen, Germantown, MD) was used to prepare the genomic library for Illumina 2 × 300-bp MiSeq sequencing. The raw reads were subjected to quality processing using Trimmomatic v0.40 to remove low-quality reads/regions and Illumina adapters. Unless otherwise stated, all software used default parameters. The reads were then assembled using Shovill (GPLV3) (https://github.com/tseemann/shovill) with the SPAdes v3.15.3 assembler and annotated using the NCBI Prokaryotic Genome Annotation Pipeline (PGAP) v5.2 (9). Following trimming, 639,894 reads corresponding to 319,947 matching paired-end reads were used for assembly. The assembly resulted in 252 contigs with an *N*_50_ value of 192,568 bases and approximately 23.5× average genome coverage. The assembled genome size was 5,288,759 bp, organized into 252 contigs with a 50.57% GC content. The strain harbors the plasmid replicons IncFIB and IncFII, identified using PlasmidFinder v2.1 (Center for Genomic Epidemiology; http://www.genomicepidemiology.org/).

Two clusters for the type 6 secretion system (T6SS) were identified in NMEC15, T6SS1 and T6SS2, using NCBI BLAST analysis of contigs. The T6SS1 cluster is 30.2 kb long, with a GC content of 52.2%. The T6SS2 cluster is 27.9 kb long, with a 52% GC content.

The resistance genes identified using ResFinder v4.1 included *sul1* (sulfonamide) and *aadA1* (aminoglycoside). The E. coli virulence factors identified through VirulenceFinder v2.0 included the increased serum survival gene (*iss*), the S-fimbria minor subunit (*sfaS*), and the vacuolating autotransporter toxin (*vat*).

### Data availability.

The genome sequence has been deposited in the National Center for Biotechnology Information (NCBI) Sequence Read Archive (SRA) under accession number SRX10988923 (NMEC15), BioProject accession number PRJNA732675, BioSample accession number SAMN19334708, and genome accession number JAHTGQ000000000.
